# Phylotranscriptomic Analyses Resolve Evolutionary History of *Eremopyrum* (Triticeae; Poaceae)

**DOI:** 10.1002/ece3.70840

**Published:** 2025-02-16

**Authors:** Shu‐Qi Fan, Hao Yan, Yue Zhang, Xiao Ma, Jun‐Ming Zhao, Hai‐Qin Zhang, Yong‐Hong Zhou, Xing Fan, Yong‐Xian Wen, Li‐Na Sha

**Affiliations:** ^1^ College of Computer and Information Science Fujian Agriculture and Forestry University Fuzhou Fujian China; ^2^ College of Grassland Science and Technology Sichuan Agricultural University Chengdu Sichuan China; ^3^ Triticeae Research Institute Sichuan Agricultural University Chengdu Sichuan China

**Keywords:** *Eremopyrum*, hybridization, introgression, polyploid, speciation

## Abstract

Disentangling the phylogenetic relationship of polyploid species is essential for understanding how such polyploid species evolved following their origin. To investigate the speciation and evolutionary history of *Eremopyrum*, we analyzed 36 transcriptomes from 9 polyploid accessions of *Eremopyrum* and 27 diploid taxa representing 12 basic genomes in Triticeae. Phylogenetic reconstruction, divergence time, and introgression event demonstrated that (1) *Eremopyrum* and *Agropyron* shared a common ancestor; (2) *Eremopyrum* has undergone ongoing evolutionary diversification since its origin in Late Miocene; (3) the diploid *E. triticeum* and *E. distans* were the genome donors of the tetraploid species of *Eremopyrum*; (4) both *Eremopyrum* and *Agropyron* contribute to the nonmonophyletic origin of tetraploid *E. orientale* via introgression events. Our results shed new light on our understanding of the diversity and ecological adaptation of the species in *Eremopyrum*.

## Graphical abstract

Disentangling the phylogenetic relationship of polyploid species is essential for understanding how such polyploid species evolved following their origin. To investigate the speciation and evolutionary history of *Eremopyrum*, we analyzed 36 transcriptomes from 9 polyploid accessions of *Eremopyrum* and 27 diploid taxa representing 12 basic genomes in Triticeae. Our results shed new light on our understanding of diversity and ecological adaptation of the species in *Eremopyrum*.

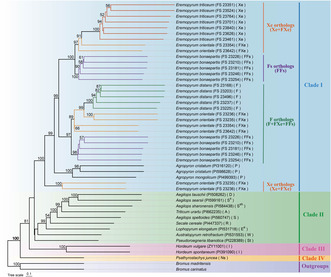

## 1 Introduction

Hybridization is a major driver of evolutionary innovation and speciation in plants (Mallet 2007; Jiao et al. 2011; Soltis and Soltis 2016; Forrester and Ashman 2018). By chromosome doubling following hybridization, polyploidization produces new genetic combination, which can stimulate changes in genome size, genomic rearrangement, gene expression, and epigenetic effect (Soltis and Soltis 2016; Otto 2007). Polyploidization can stabilize genetic combination by reducing genetic segregation and eliminating hybrid sterility, which allows polyploids to be competitively superior to the parental donor in range expansions (Otto 2007; Fan, Sha, Dong, et al. 2013; Yan and Sun 2012). Due to these benefits, 40%–70% of plants are polyploids in nature (Otto and Whitton 2000), all of which nearly 25% of vascular plants have experienced one or more episodes of polyploidization events in their ancestry (Wood et al. 2009; Li et al. 2015). It has also been considered that multiple origins of polyploid species are the rule rather than the exception (Soltis et al. 2014; Li et al. 2015; Sha et al. 2017). Since polyploidization is common, disentangling the phylogenetic relationship regarding the ancestral donor of polyploid species is essential for understanding how such polyploid species evolved following their origin.

*Eremopyrum* (Ledeb.) Jaub. et Spach, an annual genus in the wheat tribe (Poaceae: Triticeae), includes four species: *Eremopyrum distans* (C. Koch) Nevski, *Eremopyrum triticeum* (Gaertn.) Nevski, *Eremopyrum orientale* (L.) Jaub.et Spach, and *Eremopyrum bonaepartis* (Spreng.) Nevski (Frederiksen 1991). Since *Eremopyrum* belongs to a group of genera with 1 spikelet per node, it was earlier included in *Triticum* sect. *Eremopyrum* (Ledebour 1853) or *Agropyron* Gaertner (Bentham and Hooker 1883). Phylogenetic analysis based on morphology (Seberg and Frederiksen 2001) and DNA sequences (Escobar et al. 2011; Chen et al. 2020) indicated that *Eremopyrum* was related to *Agropyron*. *Eremopyrum* was first recognized as a separate genus by Jaubert and Spach (1851), who distinguished it from *Agropyron* based on the annual habit of all the species in *Eremopyrum*. Cytogenetically, *Eremopyrum* consist of the species with the **F** genome and *Agropyron* are composed of the basic **P** genome, treating them as different gene pool and rendering them genomically distinct from other taxa of the Triticeae (Löve 1984). Since then, a number of different *Eremopyrum* species that are distributed in the Mediterranean, Central Asia, and northwest of China have been described. *Eremopyrum* is a complex of diploid (2n = 2× = 14) and tetraploid (2n = 4× = 28). The genus includes two diploids, *E. distans* and *E. triticeum* , one tetraploid, *E. orientale* , and one with both diploid and tetraploid cytotypes, *E. bonaepartis* (Sakamoto 1979; Frederiksen 1991). Studies of chromosome pairing have concluded that the genomes of the three diploid species are different (Sakamoto 1979). *Eremopyrum orientale* is usually thought to be derived from the diploid parents *E. distans* and *E. triticeum* via allopolyploidization (Sakamoto 1979). For the tetraploid *E. bonaepartis* , Sakamoto (1979) suggested that it was derived from hybridization of diploid *E. distans* and *E. bonaepartis* . However, Frederiksen (1991) argued that the tetraploid *E. bonaepartis* did not originate from hybridization of diploid *E. distans* and *E. bonaepartis* , due to the lack of clear morphological identification. Yen and Yang (2013) proposed that *E. distans* possesses the **F** genome and *E. triticeum* has **Xe** genome. *E. orientale* is tetraploid with **FXe** genome, and *E. bonaepartis* has both diploid (**Fs** genome) and tetraploid (**FFs** genome) types. While cytogenetic studies add to our understanding of genetic affinity between different genome types by evidencing from meiotic pairing behavior, phylogenetic relationships among the species *Eremopyrum* remain unclear. As high similarity in morphological characteristics and sympatric distribution between *Eremopyrum* and *Agropyron*, additional unavoidable question concerns whether gene flows via introgressive hybridization reshape the evolutionary history of *Eremopyrum*.

Transcriptomic data provides abundant information on orthologous protein‐coding gene sequences per se, making them ideal tools for unraveling reticulate evolutionary history of radiation (Guo et al. 2021), demonstrating the pattern of diversification dynamics (Stull et al. 2021), and disentangling genetic mechanisms of key trait innovations (Zhang et al. 2022). Here, we sequenced and analyzed 36 transcriptomes from 21 samples of *Eremopyrum* and its affinitive species in Triticeae. Based on single‐copy nuclear gene data, we constructed phylogenetic analysis, estimated divergence times, and detected introgression events to reveal the speciation and evolutionary history of *Eremopyrum*. The objectives were (1) to identify the origin of *Eremopyrum*; (2) to investigate the interspecific relationships of the genus *Eremopyrum*; and (3) to reveal introgression events between *Eremopyrum* and *Agropyron*.

## 2 Materials and Methods

### 2.1 Plant Sampling, Transcriptome Sequencing, and *De Novo* Assembly

Twenty‐one accessions of the genus *Eremopyrum* were sampled, which included seven accessions of *E. triticeum* (**Xe** genome), five accessions of *E. distans* (**F** genome), four accessions of *E. orientale* (**FXe** genome), and five accessions of *E. bonaepartis* (**FFs** genome), with each accession representing samples collected from distinct sampling sites. This study included 21 new sequenced accessions from *Eremopyrum* (including 12 diploids and 9 tetraploids) and 15 previously sequenced diploid taxa representing 10 basic genomes in Triticeae (Table S1). In addition, *Bromus carinatus* and *Bromus madritensis* were used as outgroups.

Sequencing libraries were constructed and sequenced by the Novogene Bioinformatics Institute (Novogene, Beijing, China). For raw reads obtained from transcriptome sequencing, adapter and low‐quality reads were filtered to obtain clean reads. Clean reads were *de novo* assembled using Trinity v.2.5.1 (Grabherr et al. 2011). Then, Corset v1.05 (Davidson and Oshlack 2014) was used to cluster the transcript sequences and filter out redundant transcripts to extract one longest transcript per cluster as the unigene. Finally, the completeness of transcriptome assembly was assessed using BUSCO v.5.7.0 (Simão et al. 2015).

### 2.2 Single‐Copy Orthologous Nuclear Genes Identification

To illustrate the diploid–polyploid relationships for elucidating the evolutionary history of the genus *Eremopyrum*, we considered each subgenome in the tetraploid species as different samples for subsequent analysis. First, only diploid species were used to extracted single‐copy nuclear genes. Specifically, OrthoFinder v.2.5.5 (Emms and Kelly 2015) was used to determine single‐copy nuclear genes from 27 diploid accessions and two outgroups with default parameters. Then, for tetraploid species, we used these single‐copy sequences from diploid species to extract two corresponding sequences. Specifically, we used the single‐copy sequences from *E. distans* (**F** genome) as query sequences for a BLASTN search of assembled sequences from each tetraploid accession, using Blastn v.2.12.0 (Altschul et al. 1990) with an e‐value threshold of 1e‐5. For each single‐copy gene, the best hit was classified into the **F** subgenome, while the second‐best hit was classified into the **Xe**/**Fs** subgenome. Next, for the two corresponding sequences from each single‐copy gene per tetraploid accession, we applied CD‐HIT‐EST v.4.8.1 (Li and Godzik 2006) with the parameter “‐c 0.98” (Zhang et al. 2022) to reduce redundancy and excluded any single‐copy gene where only one sequence remained. Finally, we filtered the orthologous groups to ensure that each single‐copy nuclear gene contains 47 sequences, representing the 27 diploid accessions, two outgroups, and the two corresponding subgenomes from each of the nine tetraploid accessions. The sequences of each single‐copy nuclear gene were aligned with MAFFT v.7.505 (Katoh and Standley 2013) using the “‐auto” parameter. Regions showing poor alignment were trimmed using trimAl v.1.4 (Capella‐Gutiérrez, Silla‐Martínez, and Gabaldón 2009) with the parameter “‐automated1”.

### 2.3 Phylogenetic Analysis

Phylogenetic analysis is routinely applied to illustrate speciation and evolutionary problems. The coalescence‐based method was used to infer species trees from nucleotide sequences. For single‐copy nuclear genes, individual ML gene trees were first constructed using RAXML v.8.2.12 (Stamatakis 2014) with 100 replicates under GTRGAMMA model. Then, the best ML gene trees and 100 bootstrap replicate trees were used to estimate the coalescence‐based species tree and supporting values by ASTRAL v.5.6.3 (Mirarab et al. 2014).

### 2.4 Divergence Time Estimation

Divergence time was estimated from the coalescence‐based species tree using the program MCMCTree in the PAML package v.4.9 (Yang 2007), with *B. carinatus* and *B. madritensis* as outgroups. We used three fossil calibration points and distributed throughout the tree: (1) the root of the tree, i.e., the stem age of Triticeae set at 21.1–21.5 Mya; (2) the crown age of Triticeae set at 15.9–16.3 Mya; and (3) the stem age of *Hordeum* set at 13.4–13.7 Mya (Zhang et al. 2022). Markov chain Monte Carlo analysis was run to sample 1,000,000 times with a sampling frequency of 10 and a burn‐in of 2,000,000 iterations with the GTR + G model. In addition, two independent runs were carried out, and the effective sample sizes for all the parameters were checked in Tracer v.1.7 (Rambaut et al. 2018) to ensure they were > 200.

### 2.5 *D*_FOIL_ Analysis to Detect Introgression

*D*_FOIL_ (Pease and Hahn 2015) is widely used to infer recent and ancient introgression because it allows estimating the direction of gene flow and inferring gene flow between the ancestor of a species pair and extant species (Meleshko et al. 2021; Feng et al. 2023). In addition, this test requires an asymmetric five‐taxon tree with a specific topology (((*P*_*1*_, *P*_*2*_), (*P*_*3*_, *P*_*4*_)), Outgroups), where the divergence of (*P*_*3*_, *P*_*4*_) occurs before the divergence of *P*_*1*_ and *P*_*2*_. To detect introgression events and the direction of introgression signals between *Eremopyrum* and *Agropyron*, we used four datasets as *P*_*1*_ and *P*_*2*_ in the *D*_FOIL_ analysis. Here, *P*_*3*_, *P*_*4*_, and outgroups were consistently *A. cristatum* (**P**), *A. mongolicum* (**P**), and *Bromus* species ( *B. carinatus* and *B. madritensis* ). The four datasets used were as follows: (1) Five accessions of *E. distans* (**F**) and seven accessions of *E. triticeum* (**Xe**). We used *D*_FOIL_ with default parameters to examine the introgression signals in 17,640 individual combinations, with each combination consisting of one single‐copy nuclear sequence from one accession per species; (2) Five **F**‐orthologs and five **Fs**‐orthologs of *E. bonaepartis* , yielding a total of 12,600 combinations; (3) **Xe**‐orthologs and **F**‐orthologs from two accessions (FS 23354 and FS 23642) of *E. orientale* , resulting in 2,016 combinations; and (4) **Xe**‐orthologs and **F**‐orthologs from two accessions (FS 23235 and FS 23236) of *E. orientale* , also yielding 2 combinations. The selection of these species combinations was based on their phylogenetic relationship and ploidy levels.

## 3 Results

### 3.1 Transcriptome Sequencing, Assembly, and Single‐Copy Orthologous Identification

The transcriptome sequencing and assembly results are listed in Table S2. After filtering for adapter and low‐quality sequences of the raw reads, the clean reads per sample ranged from 18,344,392 to 23,511,708, with a mean of 20,865,901. *De novo* assembly generated transcripts ranging from 39,731 to 75,538 and the N50 of all samples was > 1702 bp. After selecting the longest transcript from each cluster to generate unigenes, the transcriptomes had an average of 30,644 unigenes and an average N50 of 1722 bp per sample. In addition, transcriptomes were assembled with an average BUSCO completeness of 88.9%.

A total of 674 single‐copy genes were identified from the unigenes of both 27 diploid samples and outgroups. These single‐copy sequences were then used to extract two corresponding sequences for tetraploid species. However, for some single‐copy genes, only one sequence was retained in tetraploid species due to random loss. After excluding these genes, *E. bonaepartis* remained 393 single‐copy genes and *E. orientale* remained 355 single‐copy genes. Next, the shared single‐copy genes were selected, resulting in a total of 296 single‐copy genes. Finally, after reducing redundancy using CD‐HIT‐EST v.4.8.1, 126 single‐copy genes remained for subsequent analysis.

### 3.2 Phylogenetic Analyses

**FIGURE 1:**
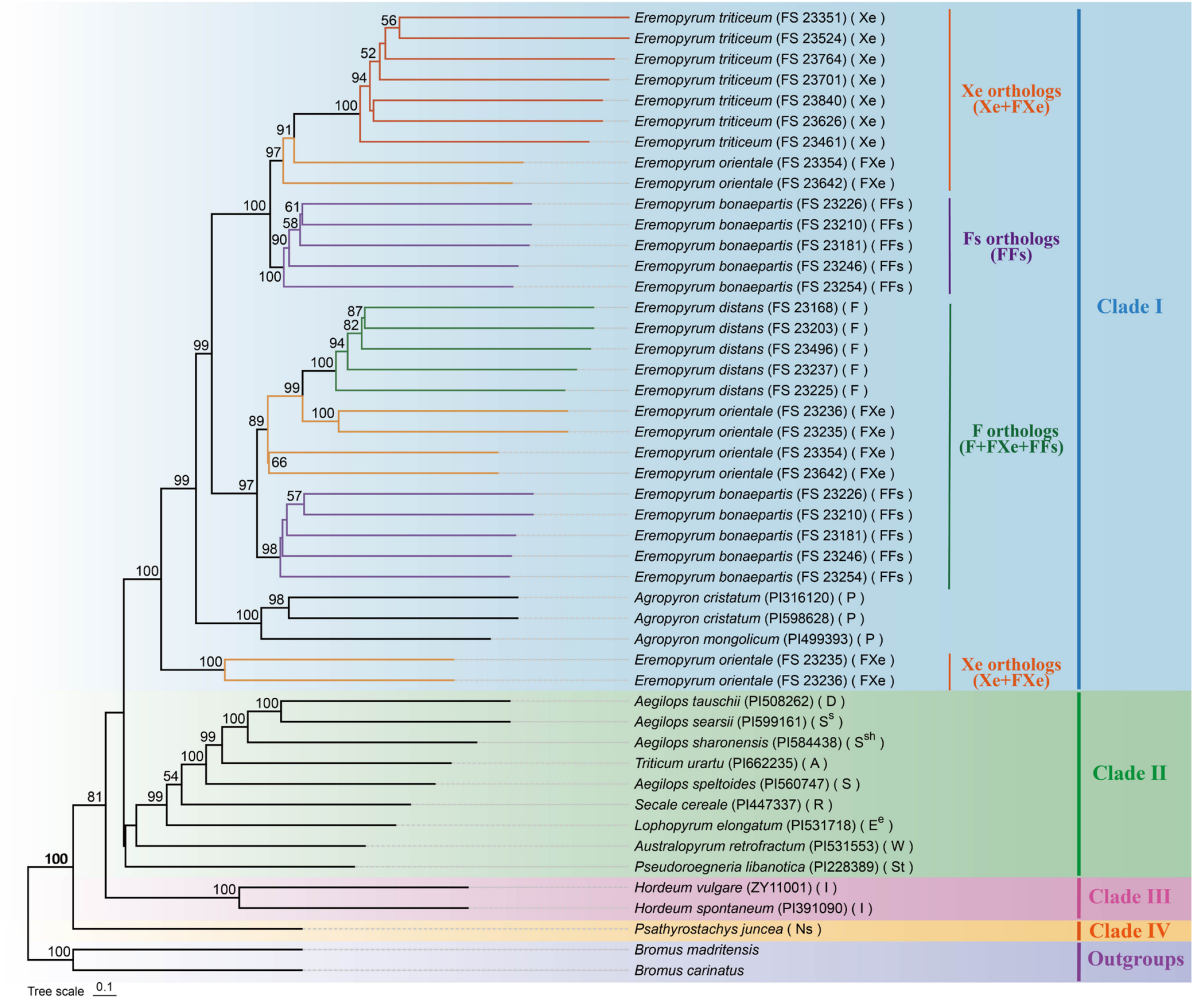
Phylogenetic tree inferred from the sequences of *Eremopyrum* species and the sequences of its affinitive species in Triticeae, under GTRGAMMA model. The numbers at the nodes indicate bootstrap values > 50%. The capital letters in the second bracket indicate the genome type of the species. Different colors labeled the branches of the *Eremopyrum* species. The tree is marked with distinct colored blocks, representing five different clades: Clade I (blue), Clade II (green), Clade III (pink), Clade IV (orange), and Clade V, i.e., outgroups (purple).

To determine the phylogenetic relationships within the genus *Eremopyrum*, phylogenetic analysis was constructed based on the 126 single‐copy genes from 21 *Eremopyrum* species and its affinitive species using the coalescence‐based method. The ML analysis generated the tree topology with BS > 50% at the nodes and all the sequences were grouped into five clades (Figure 1). Clade I contained species from *Agropyron* and *Eremopyrum*. Moreover, *Eremopyrum* species were split into three well‐supported sub‐clades related to genome orthologs (> 97% BS) (**Xe** orthologs, **Fs** orthologs, **F** orthologs), with the **Xe** orthologs subdivided into two **Xe** groups. The first group included **Xe** genome of seven accessions of *E. triticeum* and **Xe**‐orthologs of two accessions (FS 23354 and FS 23642) of *E. orientale* (97% BS). The second group of the **Xe** orthologs comprised **Xe**‐orthologs from two accessions (FS 23235 and FS23236) of *E. orientale* and located at the base of the Clade I (100% BS). **Fs** orthologs consisted of **Fs**‐orthologs from five accessions of *E. bonaepartis* (100% BS). **F** orthologs contained **F** genome of *E. distans* and **F**‐orthologs from *E. orientale* , *E. bonaepartis* (97% BS). *A. cristatum* and *A. mongolicum* of *Agropyron* were included in Clade I as a branch and were placed between **F** orthologs and the second **Xe** orthologs (100% BS). In addition, Clade II included species from *Aegilops*, *Triticum*, *Lophopyrum*, *Secale*, *Australopyrum*, and *Pseudoroegneria*, with each forming a branch. Clade III consisted of species from *Hordeum* (100% BS). Clade IV was the *Psathyrostachys* and Clade V (i.e., Outgroups) comprised species from *Bromus*.

### 3.3 Divergence Time Estimation

**FIGURE 2:**
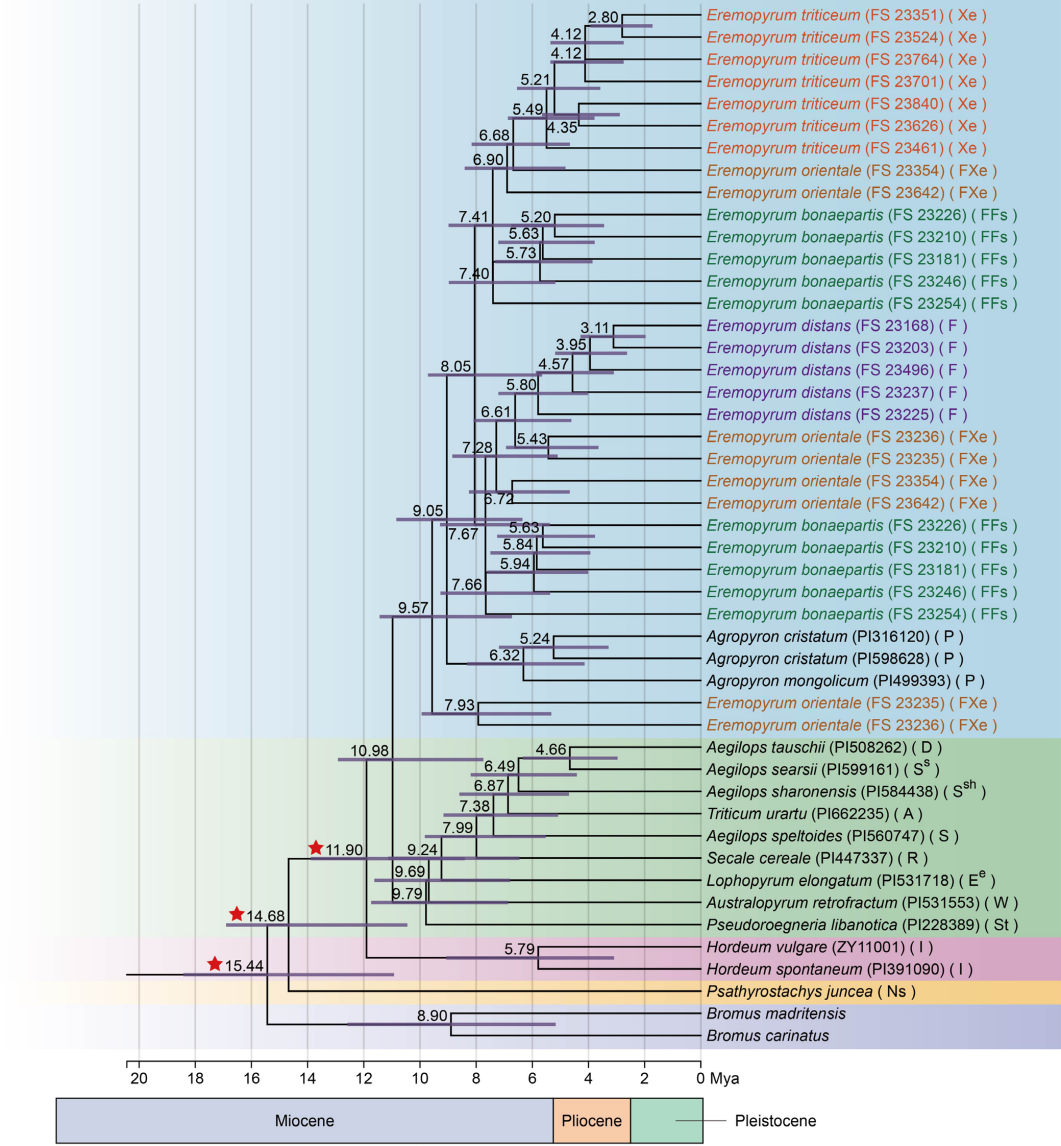
A time‐calibrated tree inferred from the sequences of *Eremopyrum* species and its affinitive species within Triticeae using MCMCTree. The ages of stratigraphic boundaries were obtained from the International Chronostratigraphic Chart (Cohen et al. 2013), with a scale as millions of years ago (Mya). Three red stars represent the three calibration points used.

On the basis of the sequence dataset containing 36 species from Triticeae, divergence times with 95% CI by using MCMCTree generated a time‐calibrated tree (Figure 2). The divergence time was marked on branch nodes within the ML tree. The time calibration analysis demonstrated that the stem age of the Triticeae was estimated to be 15.44 Mya (95% CI, 10.92–18.42), and the crown age was estimated to be 14.68 Mya (95% CI, 10.45–16.90). The maternal ancestor of *Hordeum* originated about 5.79 Mya (95% CI, 3.09–9.07). The divergence time in Clade II (*Aegilops*, *Triticum*, *Lophopyrum*, *Secale*, *Australopyrum*, and *Pseudoroegneria*) was estimated to be 9.79 Mya (95% CI, 6.86–11.74). In the time calibration tree, all the sampled *Eremopyrum* taxa formed Clade I and the genus *Agropyron* was included, and the divergence time of Clade I was 9.57 Mya (95% CI, 6.73–11.44), with the divergence time of *Agropyron* was estimated to be 9.05 Mya (95% CI, 6.35–10.84). In addition, the time calibration result indicated that the divergence time of the **Fs**‐, **F**‐, **Xe**‐genome were estimated to be 7.40 Mya (95% CI, 5.18–8.97), 7.67 Mya (95% CI, 5.37–9.29), and 9.57 Mya (95% CI, 6.73–11.44), respectively.

### 3.4 Introgression Between *Eremopyrum* and *Agropyron*

**FIGURE 3:**
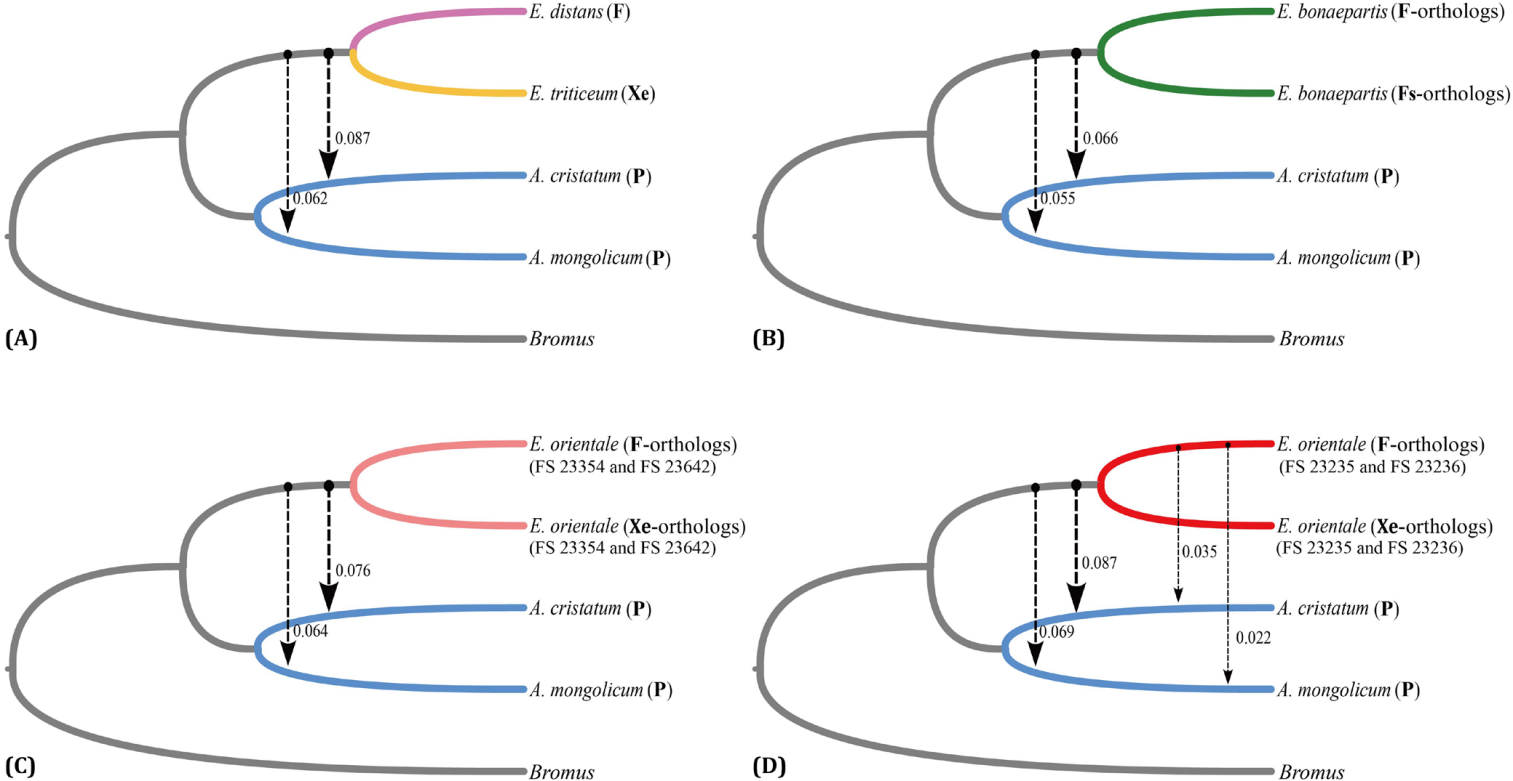
Gene flow signals were inferred between *Eremopyrum* and *Agropyron* using *D*_FOIL_ analysis, with branches representing different species indicated by different colors. Arrows depict the direction of gene flow, while the number on the right side of each arrow represents the frequency of those gene flow signals. Different thickness of the arrows signifies different gene flow frequencies.

Our *D*_FOIL_ analysis indicated that all five‐taxon combinations might undergo different level of introgression between *Eremopyrum* and *Agropyron*. Specifically, we identified eight events of ancient introgression and two events of recent introgression (Figure 3). When testing the topology including diploid *E. distans* (**F**) and diploid *E. triticeum* (**Xe**), we observed introgression signals from the ancestor of two diploid *Eremopyrum* species to both *A. cristatum* (**P**) and *A. mongolicum* (**P**). The proportions of introgression were 0.087 for *A. cristatum* and 0.062 for *A. mongolicum* , respectively (Figure 3A). Furthermore, when estimating the topology including *E. orientale* (FS 23354 and FS 23642), we detected signals from the ancestor of *E. orientale* (**F**‐orthologs) and *E. orientale* (**Xe**‐orthologs) to *A. cristatum* and *A. mongolicum* (0.076 and 0.064, respectively) (Figure 3C). When the combination of two other accessions of *E. orientale* (FS 23235 and FS 23236) was selected for *D*_FOIL_ analysis, we further found that the introgression signals were significantly strengthened, and we detected two recent signals from *E. orientale* (**F**‐orthologs) to both *A. cristatum* and *A. mongolicum* , with proportions of 0.035 and 0.022, respectively (Figure 3D). In addition, introgression signals also occurred between *Agropyron* and the ancestor of *E. bonaepartis* (**F**‐orthologs) and *E. bonaepartis* (**Fs**‐orthologs) (Figure 3B).

## 4 Discussion

In this study, we constructed a phylogenetic framework using 21 newly sequenced samples of *Eremopyrum* and 15 previously sequenced diploids to investigate the phylogenetic relationships of the genus *Eremopyrum*. Our primary strategy involved treating the different subgenomes of tetraploid species as distinct samples for phylogenetic analysis. In addition, we conducted an overall phylogenetic analysis without splitting subgenomes of tetraploid species (relevant detailed method and results are described in Data S1). After comparing the results of these two analyses, we observed that despite a high similarity in the topology of the two resulting trees, the strategy of treating the different subgenomes of tetraploid species as distinct samples provided a clearer and more intuitive reflection of the relationships between diploid species and tetraploid species within the genus *Eremopyrum*, thereby facilitating the investigation of the speciation and evolutionary history of *Eremopyrum*.

*Eremopyrum* is morphologically closely related to *Agropyron*, with especially some specific features, such as one‐keeled glumes and caryopsis morphology, being highly similar between them (Frederiksen 1991). Their close relationship has been revealed by several DNA sequence information (Petersen et al. 2006; Escobar et al. 2011; Chen et al. 2020). However, *Eremopyrum* is distinguished from *Agropyron* by its annual, self‐crossing, and short plants, with spikes that disarticulate at maturity (wedge‐type disarticulation) (Yen and Yang 2013). Intergeneric hybridizations also showed strong sterility barriers between *Eremopyrum* species and *Agropyron* (Frederiksen and von Bothmer 1995). In the present phylogenetic tree, *Agropyron* was nested into the clade including *Eremopyrum*, which is in agreement with previous molecular studies (Petersen et al. 2006; Escobar et al. 2011; Chen et al. 2020). Our time‐calibrated phylogeny further showed that *Eremopyrum* and *Agropyron* diverged at 9.57 MYA and the divergence of the **F**‐, **Fs**‐, and **Xe**‐genome lineages in *Eremopyrum* and the **P** genome lineages in *Agropyron* was dated to 9.05 MYA. Given the present data, *Eremopyrum* and *Agropyron* shared a common ancestor.

Diploid *E. triticeum* and *E. distans* are morphologically well‐defined species and often grow in mixed populations with their descendant allotetraploid species. Despite wider morphological variation, tetraploid *E. orientale* possesses some basic similarities in common with both *E. distans* and *E. triticeum* (e.g., the densely haired glumes and lemmas as in *E. distans* and the slightly hooded glumes as in *E. triticeurn*) (Sakamoto 1979; Frederiksen 1991; Yen and Yang 2013). Based on chromosome pairing behavior, Sakamoto (1979) proposed that *E. orientale* is an allopolyploid involving *E. distans* and *E. triticeum* . In this study, **F** orthologs contained diploid *E. distans* and tetraploid *E. orientale* and *E. bonaepartis*. The plastome data also suggest that *E. distans* was the maternal ancestor of the tetraploid species during the hybridization processes (relevant detailed methods and results are described in Data S2). Orthologs of two sampled accessions (FS 23354 and FS 23642) of *E. orientale* were split into two well‐supported clades, which separately correspond to the **Xe** genome of *E. triticeum* and the **F** genome of *E. distans* . This confirms that *E. orientale* originated from hybridization between *E. triticeum* and *E. distans* . For the two sampled accessions (FS 23235 and FS 23236) of *E. orientale* , one orthologous type (**F**‐orthologs) were grouped with *E. distans* and the other type (modified **Xe**‐orthologs) was placed outside the clade including *Eremopyrum* and *Agropyron*. These results indicate a nonmonophyletic origin of *E. orientale* , which associated with introgression events after polyploidization. In most cases, the distribution of the tetraploid species overlaps, completely or partly, with that of their putative diploid parents as well as *Agropyron*, providing physical proximity for introgression events. Our observed different frequency and strength of gene flow between *Agropyron* and *Eremopyrum* indicated the occurrence of intragenic and intergenic introgression events. Because frequent gene flow events usually reduce the genetic differences between the species, greater strength of gene flow between the first two accessions of *E. orientale* and *E. bonaepartis* and between the last two accessions of *E. orientale* and *Agropyron* would largely contribute to the nonmonophyletic origin of *E. orientale* . Different accessions of *E. orientale* thus can be shown at different clades of a phylogenetic tree, with the first two accessions being more closely related to the species of *Eremopyrum* and the last two accessions being more closely related to *Agropyron*.

Estimating the divergence times of the lineages of the genus *Eremopyrum* helps us to understand the causes of speciation within the genus *Eremopyrum*. Divergence time results show that *Eremopyrum* may have originated in the Late Miocene and have undergone ongoing evolutionary diversification during the period of 2.80–9.57 Mya. Plant species diversification has been found to be highly correlated with climate change (Jaramillo, Rueda, and Mora 2006; Hoorn et al. 2010; Fan, Sha, Yu, et al. 2013). It is reported that the global climate became more arid and atmospheric CO_2_ levels decreased after the Middle Miocene climatic optimum (16.8–14.7 Mya) (Holbourn et al. 2014), contributing to the rise of global dryland plants. These climatic changes may have led to increased aridity in Central Asia, of which northern Xinjiang is a part. Such new ecological habitat, together with interspecific hybridization, promotes speciation and adaptation of *Eremopyrum*, thereby increasing the diversity of *Eremopyrum* species.

In conclusion, this study sheds light on the phylogenetic relationship, divergence time, and introgression event. These results provide valuable insights into the speciation and evolutionary history of *Eremopyrum*. Moreover, the transcriptomic data and analytical methods in this study offer important resources for further research on *Eremopyrum* species. Unfortunately, due to the difficulty in sampling diploid *E. bonaepartis* , data from this species were not included in this study. In the future, we will address the limitation and continue to investigate the phylogenetic relationships and evolutionary history of *Eremopyrum* to enhance the efficiency for the utility and conservation of plant germplasm resources.

## Author Contributions

**Shu‐Qi Fan:** investigation (equal), methodology (equal), writing – original draft (lead). **Hao Yan:** investigation (equal), methodology (equal). **Yue Zhang:** investigation (equal), methodology (equal). **Xiao Ma:** investigation (equal), methodology (equal). **Jun‐Ming Zhao:** investigation (equal), methodology (equal). **Hai‐Qin Zhang:** investigation (equal), methodology (equal). **Yong‐Hong Zhou:** conceptualization (equal), writing – review and editing (equal). **Xing Fan:** conceptualization (equal), writing – review and editing (equal). **Yong‐Xian Wen:** writing – review and editing (equal). **Li‐Na Sha:** conceptualization (equal), funding acquisition (lead), writing – review and editing (equal).

## Conflicts of Interest

The authors declare no conflicts of interest.

## Supporting information


**Table S1** List of taxa used in this study.

**Table S2** Information of transcriptomes included in this study.

**Data S1** Phylogenetic tree inferred from the sequences of *Eremopyrum* species (subgenomes of tetraploid *Eremopyrum* have not been split).

**Data S2** Phylogenetic tree inferred from the plastome sequences of *Eremopyrum* species.

## Data Availability

The raw sequence data used in this study have been deposited in the Genome Sequence Archive (Genomics, Proteomics & Bioinformatics 2021) in National Genomics Data Center (Nucleic Acids Res 2022), China National Center for Bioinformation/Beijing Institute of Genomics, Chinese Academy of Sciences (GSA: CRA017277) that are publicly accessible at https://ngdc.cncb.ac.cn/gsa.
