# Supplementary material for: Phylotranscriptomic Analyses Resolve Evolutionary History of Eremopyrum (Triticeae; Poaceae)
Source: Ecol Evol. 2025 Feb 16;15(2):e70840. doi: 10.1002/ece3.70840 (PMC11830566; doi:10.1002/ece3.70840)
Supplement: Supplementary file 1 — Table S1 List of taxa used in this study. [file ECE3-15-e70840-s001.docx]

**Table S1** List of taxa used in this study

| **Species** | **Accession No.** | **Genome** | **Ploidy** | **Origin** |
| --- | --- | --- | --- | --- |
| ***Aegilops* L.** |  |  |  |  |
| *Aegilops searsii* | PI599161 | S^b^ | 2x | HaDarom, Israel |
| *Aegilops sharonensis* | PI584438 | S^sh^ | 2x | HaMerkaz, Israel |
| *Aegilops speltoides* | PI560747 | S | 2x | Turkey |
| *Aegilops tauschii* | PI508262 | D | 2x | China |
| ***Agropyron* J. Gaertn.** |  |  |  |  |
| *Agropyron cristatum* | PI598628 | P | 2x | Kazakhstan |
| *Agropyron cristatum* | PI316120 | P | 2x | Australia |
| *Agropyron mongolicum* | PI499393 | P | 2x | Inner Mongolia, China |
| ***Australopyrum* (Tzvelev) Á. Löve** |  |  |  |  |
| *Australopyrum retrofractum* | PI531553 | W | 2x | Australia |
| ***Eremopyrum* (Ledeb.) Jaub. & Spach** |  |  |  |  |
| *Eremopyrum bonaepartis* | FS 23210 | FFs | 4x | Xinjiang, China |
| *Eremopyrum bonaepartis* | FS 23181 | FFs | 4x | Xinjiang, China |
| *Eremopyrum bonaepartis* | FS 23246 | FFs | 4x | Xinjiang, China |
| *Eremopyrum bonaepartis* | FS 23226 | FFs | 4x | Xinjiang, China |
| *Eremopyrum bonaepartis* | FS 23254 | FFs | 4x | Xinjiang, China |
| *Eremopyrum distans* | FS 23168 | F | 2x | Xinjiang, China |
| *Eremopyrum distans* | FS 23496 | F | 2x | Xinjiang, China |
| *Eremopyrum distans* | FS 23237 | F | 2x | Xinjiang, China |
| *Eremopyrum distans* | FS 23225 | F | 2x | Xinjiang, China |
| *Eremopyrum distans* | FS 23203 | F | 2x | Xinjiang, China |
| *Eremopyrum orientale* | FS 23354 | FXe | 4x | Xinjiang, China |
| *Eremopyrum orientale* | FS 23236 | FXe | 4x | Xinjiang, China |
| *Eremopyrum orientale* | FS 23235 | FXe | 4x | Xinjiang, China |
| *Eremopyrum orientale* | FS 23642 | FXe | 4x | Xinjiang, China |
| *Eremopyrum triticeum* | FS 23351 | Xe | 2x | Xinjiang, China |
| *Eremopyrum triticeum* | FS 23461 | Xe | 2x | Xinjiang, China |
| *Eremopyrum triticeum* | FS 23764 | Xe | 2x | Xinjiang, China |
| *Eremopyrum triticeum* | FS 23701 | Xe | 2x | Xinjiang, China |
| *Eremopyrum triticeum* | FS 23626 | Xe | 2x | Xinjiang, China |
| *Eremopyrum triticeum* | FS 23524 | Xe | 2x | Xinjiang, China |
| *Eremopyrum triticeum* | FS 23840 | Xe | 2x | Xinjiang, China |
| ***Hordeum* L.** |  |  |  |  |
| *Hordeum spontaneum* | PI391090 | I | 2x | Hefa, Israel |
| *Hordeum vulgare* | ZY11001 | I | 2x | China |
| ***Lophopyrum* (Host) Á. Löve** |  |  |  |  |
| *Lophopyrum elongatum* | PI531718 | E^e^ | 2x | Tunisia |
| ***Psathyrostachys* Nevski** |  |  |  |  |
| *Psathyrostachys juncea* | -- | Ns | 2x | NA |
| ***Pseudoroegneria* (Nevski) Á. Löve** |  |  |  |  |
| *Pseudoroegneria libanotica* | PI228389 | St | 2x | Iran |
| ***Secale* L*.*** |  |  |  |  |
| *Secale cereale* | PI447337 | R | 2x | Xinjiang, China |
| ***Triticum* L.** |  |  |  |  |
| *Triticum urartu* | PI662235 | A | 2x | Iran |
| ***Bromus* L.** |  |  |  |  |
| *Bromus carinatus* | -- | -- | -- | NA |
| *Bromus madritensis* | -- | -- | -- | NA |

NA: not available
