# Supplementary material for: Phylotranscriptomic Analyses Resolve Evolutionary History of Eremopyrum (Triticeae; Poaceae)
Source: Ecol Evol. 2025 Feb 16;15(2):e70840. doi: 10.1002/ece3.70840 (PMC11830566; doi:10.1002/ece3.70840)
Supplement: Supplementary file 2 — Table S2 Information of transcriptomes included in this study. [file ECE3-15-e70840-s002.docx]

**Table S2** Results of transcriptome sequencing and assembly in this study

| **Species** | **Accession No.** | **raw reads** | **clean reads** | **GC percent of clean data (%)** | **Sequence assembled by Trinity** | | | | **Unigene** | | | | **Complete BUSCOs (%)** |
| --- | --- | --- | --- | --- | --- | --- | --- | --- | --- | --- | --- | --- | --- |
|  |  |  |  |  | **Numbers** | **N50** | **Average length** | **Median length** | **Numbers** | **N50** | **Average length** | **Median length** |  |
| *Eremopyrum bonaepartis* | FS 23210 | 22753390 | 20898369 | 55.94 | 70792 | 1747 | 1301 | 1054 | 35023 | 1631 | 1169 | 863 | 89.3 |
| *Eremopyrum bonaepartis* | FS 23181 | 23641455 | 23511708 | 55.79 | 73356 | 1790 | 1331 | 1081 | 34859 | 1670 | 1200 | 897 | 90.3 |
| *Eremopyrum bonaepartis* | FS 23246 | 22486842 | 22361837 | 56.14 | 70455 | 1839 | 1362 | 1108 | 34277 | 1704 | 1220 | 905 | 91 |
| *Eremopyrum bonaepartis* | FS 23226 | 20929781 | 20826634 | 55.64 | 66865 | 1819 | 1368 | 1129 | 32724 | 1695 | 1240 | 947 | 91.1 |
| *Eremopyrum bonaepartis* | FS 23254 | 21354295 | 21251719 | 55.09 | 75444 | 1774 | 1314 | 1067 | 35812 | 1651 | 1179 | 866 | 90.5 |
| *Eremopyrum distans* | FS 23168 | 20844419 | 20681890 | 56.98 | 42424 | 1801 | 1334 | 1078 | 27990 | 1792 | 1333 | 1067 | 89.3 |
| *Eremopyrum distans* | FS 23496 | 21642108 | 19709822 | 55.21 | 42421 | 1842 | 1371 | 1107 | 27559 | 1829 | 1370 | 1109 | 90.7 |
| *Eremopyrum distans* | FS 23237 | 22363560 | 22258541 | 56.93 | 43406 | 1786 | 1321 | 1055 | 28193 | 1778 | 1319 | 1048 | 87.8 |
| *Eremopyrum distans* | FS 23225 | 20409802 | 18736790 | 56.46 | 40181 | 1702 | 1249 | 991 | 27502 | 1712 | 1263 | 997 | 88 |
| *Eremopyrum distans* | FS 23203 | 20372664 | 18344392 | 56.29 | 40043 | 1797 | 1347 | 1097 | 26694 | 1802 | 1356 | 1100 | 90.8 |
| *Eremopyrum orientale* | FS 23354 | 18853999 | 18725510 | 56.59 | 59904 | 1782 | 1338 | 1096 | 30459 | 1663 | 1222 | 934 | 87.4 |
| *Eremopyrum orientale* | FS 23236 | 19816888 | 19708438 | 55.66 | 74144 | 1755 | 1304 | 1057 | 35731 | 1586 | 1155 | 854 | 90.8 |
| *Eremopyrum orientale* | FS 23235 | 21544711 | 21437128 | 56.29 | 75538 | 1766 | 1280 | 1005 | 37692 | 1568 | 1122 | 800 | 87.8 |
| *Eremopyrum orientale* | FS 23642 | 23126867 | 21040312 | 54.89 | 65368 | 1794 | 1328 | 1074 | 32111 | 1678 | 1193 | 882 | 90.2 |
| *Eremopyrum triticeum* | FS 23351 | 23234681 | 21474941 | 56.95 | 39731 | 1779 | 1288 | 991 | 25138 | 1726 | 1239 | 935 | 77.5 |
| *Eremopyrum triticeum* | FS 23461 | 23688519 | 21319718 | 56.06 | 43652 | 1789 | 1327 | 1065 | 28766 | 1794 | 1338 | 1077 | 90.8 |
| *Eremopyrum triticeum* | FS 23764 | 22647681 | 20625957 | 57.31 | 43446 | 1789 | 1317 | 1039 | 27204 | 1746 | 1291 | 1013 | 82.9 |
| *Eremopyrum triticeum* | FS 23701 | 22884730 | 22772506 | 56.06 | 45377 | 1810 | 1339 | 1067 | 29272 | 1802 | 1331 | 1058 | 91.8 |
| *Eremopyrum triticeum* | FS 23626 | 23355355 | 21294521 | 55 | 46813 | 1776 | 1301 | 1038 | 28925 | 1785 | 1297 | 1019 | 91.7 |
| *Eremopyrum triticeum* | FS 23524 | 21104636 | 19632804 | 56.47 | 42269 | 1766 | 1302 | 1030 | 28186 | 1761 | 1297 | 1021 | 87.8 |
| *Eremopyrum triticeum* | FS 23840 | 21678198 | 21570391 | 56.67 | 46447 | 1841 | 1356 | 1082 | 29401 | 1802 | 1335 | 1056 | 89.7 |
