# Supplementary material for: Phylotranscriptomic Analyses Resolve Evolutionary History of Eremopyrum (Triticeae; Poaceae)
Source: Ecol Evol. 2025 Feb 16;15(2):e70840. doi: 10.1002/ece3.70840 (PMC11830566; doi:10.1002/ece3.70840)
Supplement: Supplementary file 3 — Data S1 Phylogenetic tree inferred from the sequences of Eremopyrum species (subgenomes of tetraploid Eremopyrum have not been split). [file ECE3-15-e70840-s003.docx]

**Method**

**Single-copy orthologous nuclear genes identification and phylogenetic analysis (without splitting subgenomes of tetraploid species)**

Single-copy nuclear genes were determined from 38 accessions, using OrthoFinder v.2.5.5 (Emms & Kelly, 2015) with the default parameters. The resulting 582 single-copy nuclear gene were aligned with MAFFT v.7.505 (Katoh & Standley, 2013) using the “-auto” parameter. Regions showing poor alignment were trimmed using trimAl v.1.4 (Capella-Gutiérrez et al., 2009) with the parameter “-automated1”.

The coalescence-based method was used to infer species trees from nucleotide sequences. For each single-copy nuclear genes, individual ML gene trees were first constructed using RAXML v.8.2.12 (Stamatakis, 2014) with 100 replicates under GTRGAMMA model. Then, the best ML gene trees and 100 bootstrap replicate trees were used to estimate the coalescence-based species tree and supporting values by ASTRAL v.5.6.3 (Mirarab et al., 2014).

**Result**

**Phylogenetic analyses (without splitting subgenomes of tetraploid species)**

We also conducted phylogenetic analysis using 582 single-copy genes without splitting subgenomes of tetraploid species, aiming to clarity the overall phylogenetic relationships within the genus *Eremopyrum*. The ML analysis generated the tree topolopy with BS > 50 % at the nodes, and all the sequences were grouped into seven clades (Figure S1). Clade I contained species from *Agropyron* and *Eremopyrum*. Furthermore, seven accessions of *E. triticeum* (**Xe**) (100 % BS), five accessions of *E. bonaepartis* (**FFs**) (100 % BS) and five accessions of *E. distans* (**F**) (100 % BS) each formed distinct subclade. It is noteworthy that four accessions of same species, *E. orientale* (**FXe**), did not grouped into a subclade. The first two accessions (FS 23354 and FS 23642) of *E. orientale* were positioned between the subclade containing *E. triticeum* and the one consisting of *E. bonaepartis,* while the remain accession (FS 23235 and FS 23236) were placed at the base of Clade I.

**

**

**Figure S1** Phylogenetic tree inferred from the sequences of *Eremopyrum* species and the sequences of its affinitive species in Triticeae, under GTRGAMMA model, without splitting subgenomes of tetraploid species. The numbers at the nodes indicate bootstrap values > 50 %. Different colors labeled the branches of the *Eremopyrum* species.

**References**

Emms, D. M., & Kelly, S. (2015). OrthoFinder: solving fundamental biases in whole genome comparisons dramatically improves orthogroup inference accuracy. *Genome Biology, 16*, 1-14.

Katoh, K., & Standley, D. M. (2013). MAFFT multiple sequence alignment software version 7: improvements in performance and usability. *Molecular Biology and Evolution, 30*(4), 772-780.

Capella-Gutiérrez, S., Silla-Martínez, J. M., & Gabaldón, T. (2009). trimAl: a tool for automated alignment trimming in large-scale phylogenetic analyses. *Bioinformatics, 25*(15), 1972-1973.

Mirarab, S., Reaz, R., Bayzid, M. S., Zimmermann, T., Swenson, M. S., & Warnow, T. (2014). ASTRAL: genome-scale coalescent-based species tree estimation. *Bioinformatics, 30*(17), i541-i548.

Stamatakis, A. (2014). RAxML version 8: a tool for phylogenetic analysis and post-analysis of large phylogenies. *Bioinformatics*, *30*(9), 1312-1313.
