# Supplementary material for: Phylotranscriptomic Analyses Resolve Evolutionary History of Eremopyrum (Triticeae; Poaceae)
Source: Ecol Evol. 2025 Feb 16;15(2):e70840. doi: 10.1002/ece3.70840 (PMC11830566; doi:10.1002/ece3.70840)
Supplement: Supplementary file 4 — Data S2 Phylogenetic tree inferred from the plastome sequences of Eremopyrum species. [file ECE3-15-e70840-s004.docx]

**Method**

**Plastome sequences extraction from transcriptomes, construct plastome tree**

To extract plastome sequences from our transcriptomes for constructing a maternally inherited plastome tree. We first downloaded the chloroplast genome of *Eremopyrum triticeum* (GenBank MH285852.1) from NCBI and extracted the coding sequences (CDS). We used the 76 CDS of *E. triticeum* as query sequences for a BLASTN search of the unigenes from all samples in this study, using Blastn v.2.12.0 (Altschul et al., 1990) with an e-value threshold of 1e-5. Any CDS that did not match all samples was discarded. Then, the best hits were retained, and the corresponding sequences were extracted from the unigenes. For each CDS, the sequences from 38 unigenes were aligned using MAFFT v.7.505 (Katoh & Standley, 2013) with the “-auto” parameter. Finally, we combined all sequences into a matrix and regions showing poor alignment were trimmed using trimAl v.1.4 (Capella-Gutiérrez et al., 2009).

The best fitting model used for sequences matrix was determined using ModelTest-NG (Darriba et al., 2020) with Bayesian Information Criterion (BIC). The species tree was constructed using RAxML-NG (Kozlov et al., 2019) with 100 replicates under GTR+I+G4 model.

**Result**

**Phylogenetic analyses**

Plastome sequences were extracted from transcriptomes to construct a maternally inherited plastome tree. The tree illustrated in Figure S2 was the ML tree with bootstrap support (BS) above 50 % at the notes. The tree resulted into five clades (Clade I-VI). Clade I (> 90 % BS) was sister to the remaining clades, and included all accessions of the genus *Eremopyrum* and *Agropyron* species, where *Agropyron* species form a subclade, and *Eremopyrum* form a large subclade. Within the *Eremopyrum* branch, seven accessions of *E. triticeum* (**Xe**) grouped together into a branch (> 93 % BS) and diploid *E. distans* and tetraploid *E. orientale* and *E. bonaepartis* in the same subclade (> 57 % BS), but the five accessions of *E. distans* (**F**), four accessions of *E. orientale* (**FXe**), and five accessions of *E. bonaepartis* (**FFs**) did not each form a clade.

***
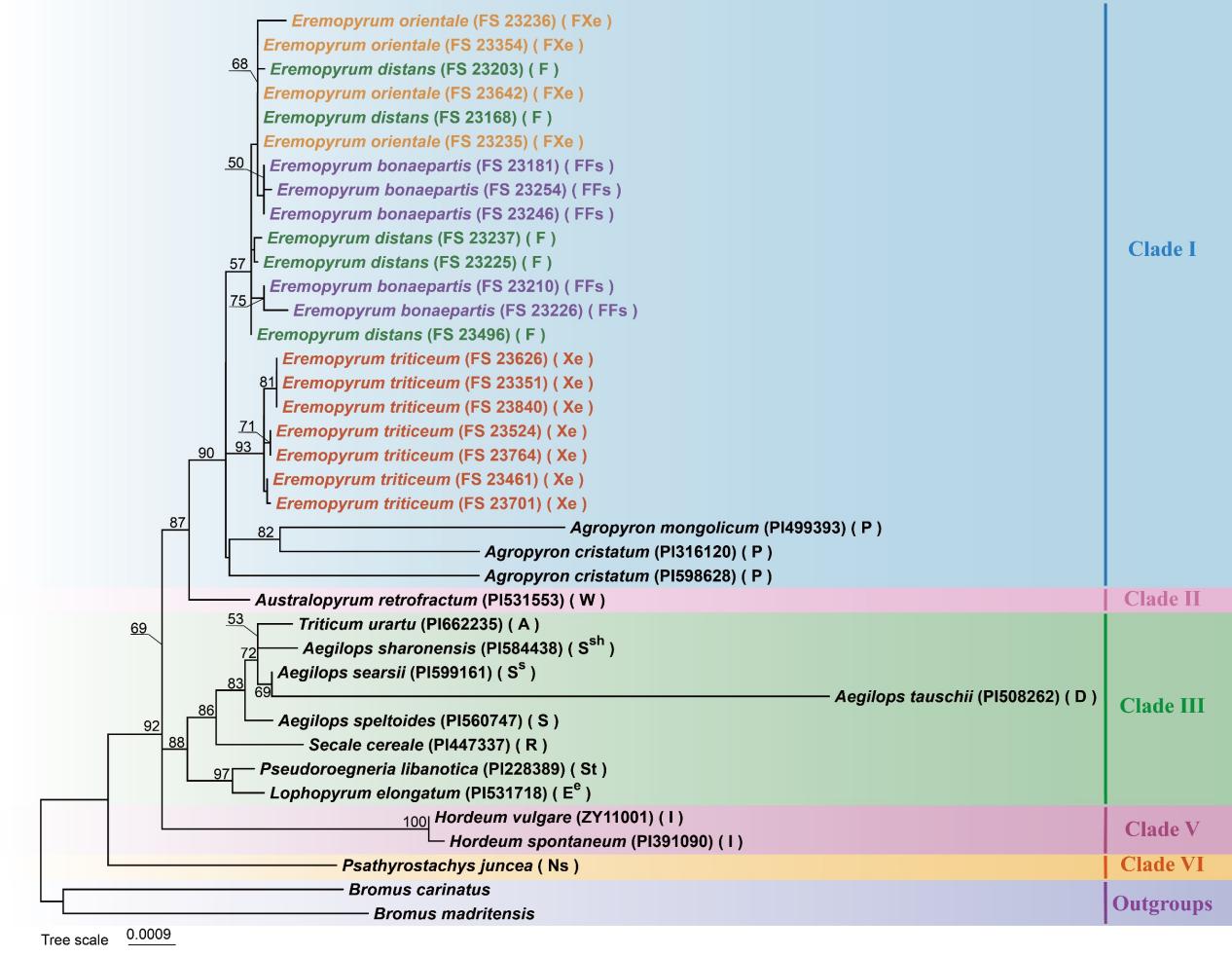
***

**Figure S2** Phylogenetic tree inferred from the plastome sequences of *Eremopyrum* species and the sequences of its affinitive species in Triticea. The numbers at the nodes indicate bootstrap values > 50 %. Different colors labeled the branches of the *Eremopyrum* species.

**References**

Altschul, S. F., Gish, W., Miller, W., Myers, E. W., & Lipman, D. J. (1990). Basic local alignment search tool. *Journal of Molecular Biology, 215*(3), 403-410.

Katoh, K., & Standley, D. M. (2013). MAFFT multiple sequence alignment software version 7: improvements in performance and usability. *Molecular Biology and Evolution, 30*(4), 772-780.

Capella-Gutiérrez, S., Silla-Martínez, J. M., & Gabaldón, T. (2009). trimAl: a tool for automated alignment trimming in large-scale phylogenetic analyses. *Bioinformatics, 25*(15), 1972-1973.

Darriba, D., Posada, D., Kozlov, A. M., Stamatakis, A., Morel, B., & Flouri, T. (2020). ModelTest-NG: a new and scalable tool for the selection of DNA and protein evolutionary models. *Molecular Biology and Evolution*, *37*(1), 291-294.

Kozlov, A. M., Darriba, D., Flouri, T., Morel, B., & Stamatakis, A. (2019). RAxML-NG: a fast, scalable and user-friendly tool for maximum likelihood phylogenetic inference. *Bioinformatics*, *35*(21), 4453-4455.
